# Supplementary material for: A method for quantitatively separating the piezoelectric component from the as-received “Piezoelectric” signal
Source: Nat Commun. 2022 Mar 16;13:1391. doi: 10.1038/s41467-022-29087-w (PMC8927587; doi:10.1038/s41467-022-29087-w)
Supplement: Supplementary file 1 — Supplementary Information [file 41467_2022_29087_MOESM1_ESM.pdf]

Supplementary Information

A Method for Quantitatively Separating the Piezoelectric Component from the as-received "Piezoelectric" Signal

Chaojie Chen [1], Shilong Zhao [1], Caofeng Pan [2], Yunlong Zi [3], Fangcheng Wang [1], Cheng Yang [1*], Zhong Lin Wang [2,4*]

[1] Institute of Materials Research, Tsinghua Shenzhen International Graduate School, Tsinghua University, Shenzhen 518055, P. R. China

[2] CAS Center for Excellence in Nanoscience, Beijing Key Laboratory of Micro-nano Energy and Sensor, Beijing Institute of Nanoenergy and Nanosystems, Chinese Academy of Sciences, Beijing 100083, P. R. China

[3] Department of Mechanical and Automation Engineering, The Chinese University of Hong Kong Shatin, N.T. Hong Kong, China

[4] School of Materials Science and Engineering, Georgia Institute of Technology, Atlanta, GA 30332-0245, USA.

Corresponding authors

[*] E-mail: yang.cheng@sz.tsinghua.edu.cn；zhong.wang@mse.gatech.edu

Supplementary Figures

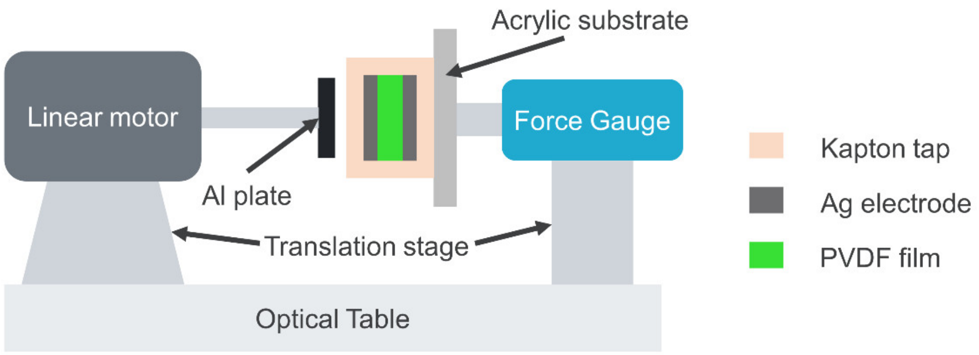

Supplementary Fig. 1 Schematic of the compression test platform including linear motor, Al plate, PVDF-based device, acrylic substrate, and force gauge.

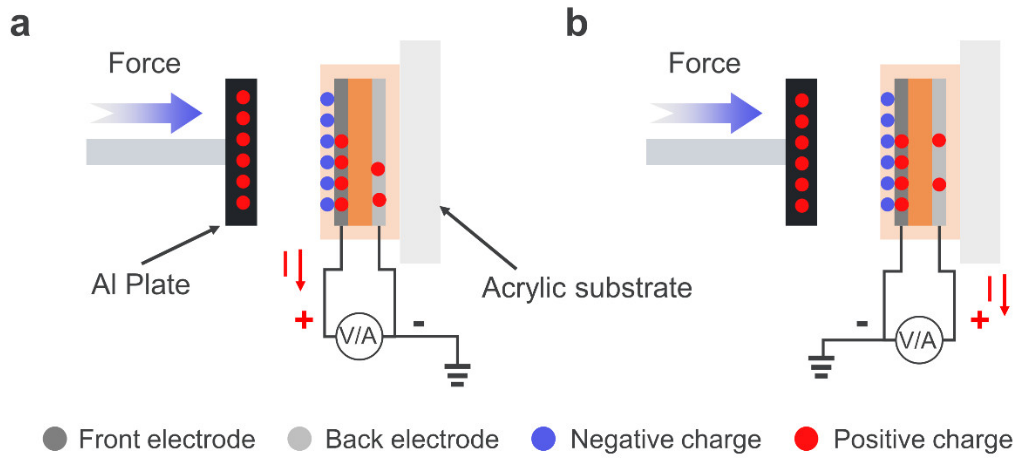

Supplementary Fig. 2 Schematic diagram of measuring the electric signal between the front electrode and back electrode of the PI-based device. (a) In the forward connection, the Kapton layer and front electrode form a SE-TENG system, and most of the induced positive charges can be detected in the front electrode. (b) In the reverse connection, most of the induced positive charges in the front electrode flow to the ground, while lesser positive charges in the back electrode are detected.

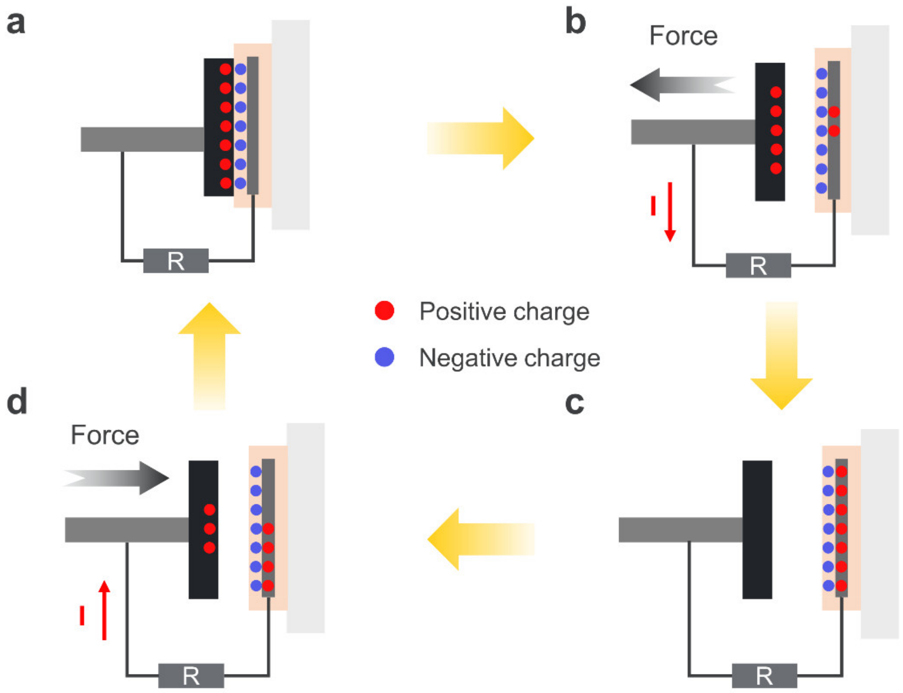

Supplementary Fig. 3 The working mechanism of the CS-TENG of the PI-based device. (a) When the Al plate and the device are contacted with each other, the same amount of positive and negative charges can be induced in the surface of the Al plate and Kapton layer respectively. (b) When the Al plate is separating from the device, the positive charges in the Al plate flow to the electrode of the device. (c) When the Al plate is well separated with the device, there is an electrostatic balance between the Kapton layer and the electrode. (d) When the Al plate is contacting with the device, the positive charges in the electrode flow to the Al plate.

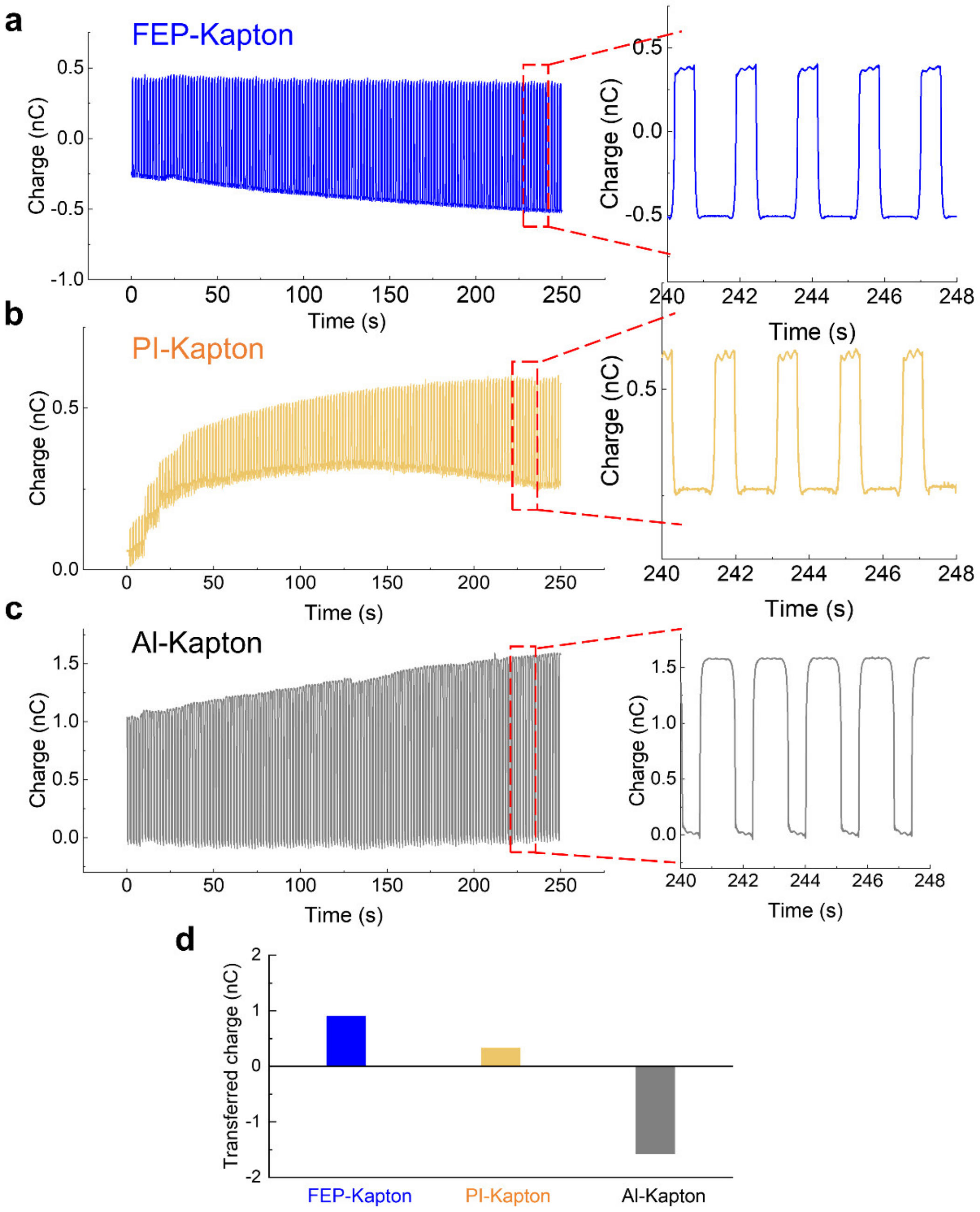

Supplementary Fig. 4 The influence of different compressing materials on triboelectric signal. The PI-based device is compressed by (a) FEP, (b) PI film, and (c) Al, resulting in different charge curves. (d) The transferred charge of FEP-Kapton is about 1 nC, that of PI-Kapton is ~0.3 nC, and that of Al-Kapton is -1.5 nC. Since these pressing materials have different electron affinities, the transferred charges have different directions and values.

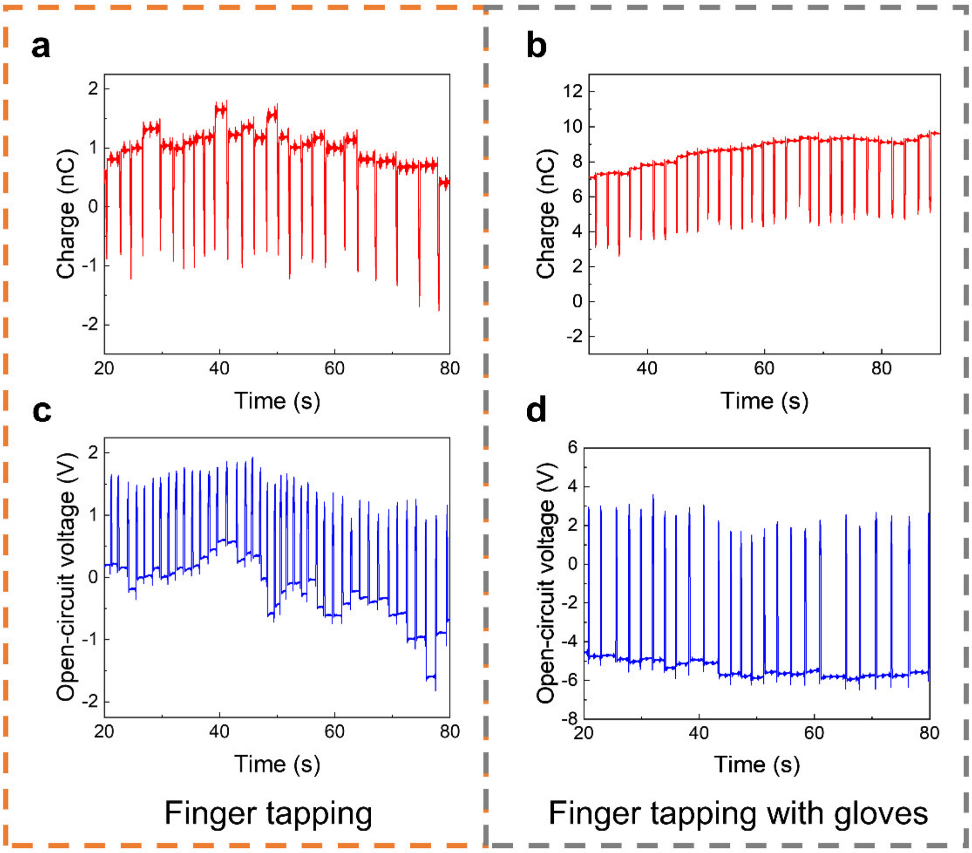

Supplementary Fig. 5 The electric response of PI-based device under finger tapping in practical measurement. The (a) transferred charge and (c) open-circuit voltage are generated when using a finger to tap the PI-based device, they are up to ~-2 nC and ~1.5 V respectively. The (b) transferred charge and (d) open-circuit voltage are generated when using a finger with a rubber glove to tap the PI-based device, they are up to ~-4 nC and ~7 V respectively.

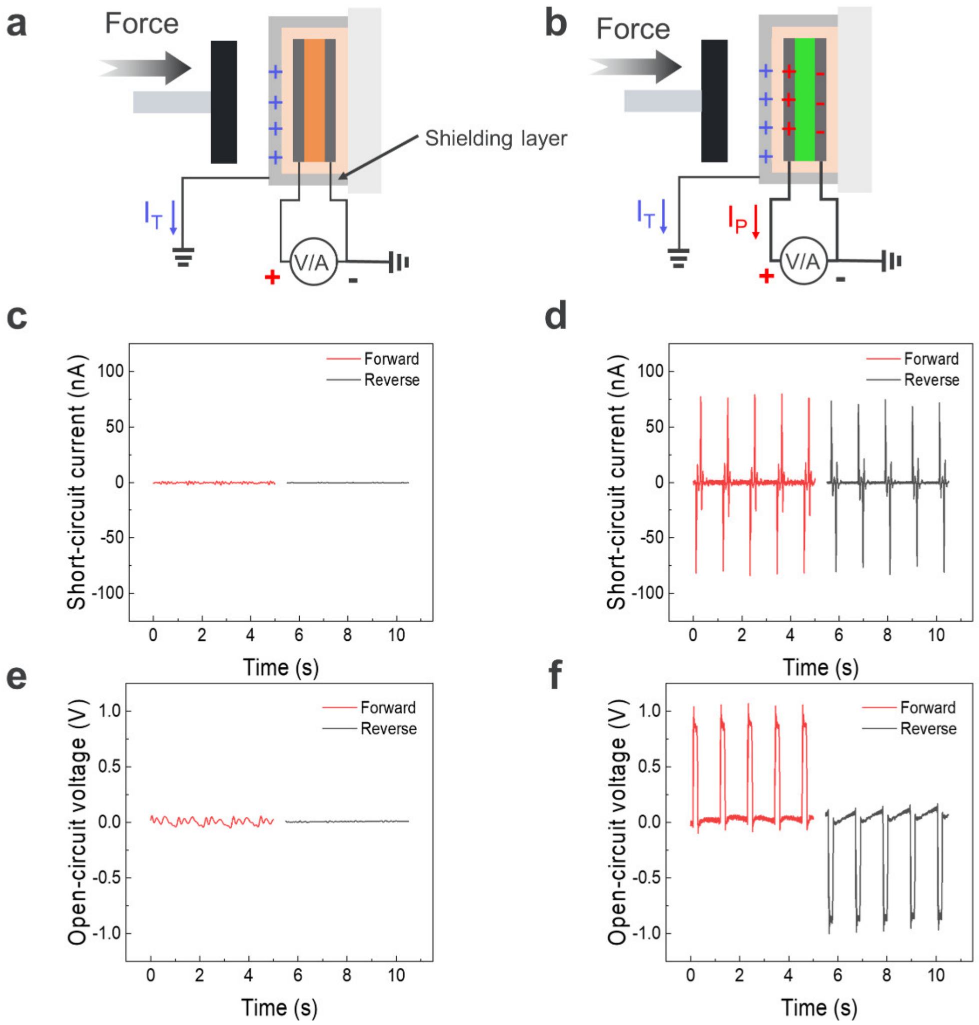

Supplementary Fig. 6 The role of conductive shielding layer. (a) Schematic diagram of measuring the electric signals from the PI-based device with a conductive shielding layer, the corresponding (c) short-circuit current and (e) open-circuit voltage curves show no electric signals in both the forward and reverse connection, indicating that the contact electrification outside the device is eliminated. (b) Schematic diagram of measuring the electric signals from the PVDF-based device with a conductive shielding layer, the corresponding (c) short-circuit current and (e) open-circuit voltage curves show significant electric signals in both the forward and reverse connection, suggesting that they are sole piezoelectric signals.

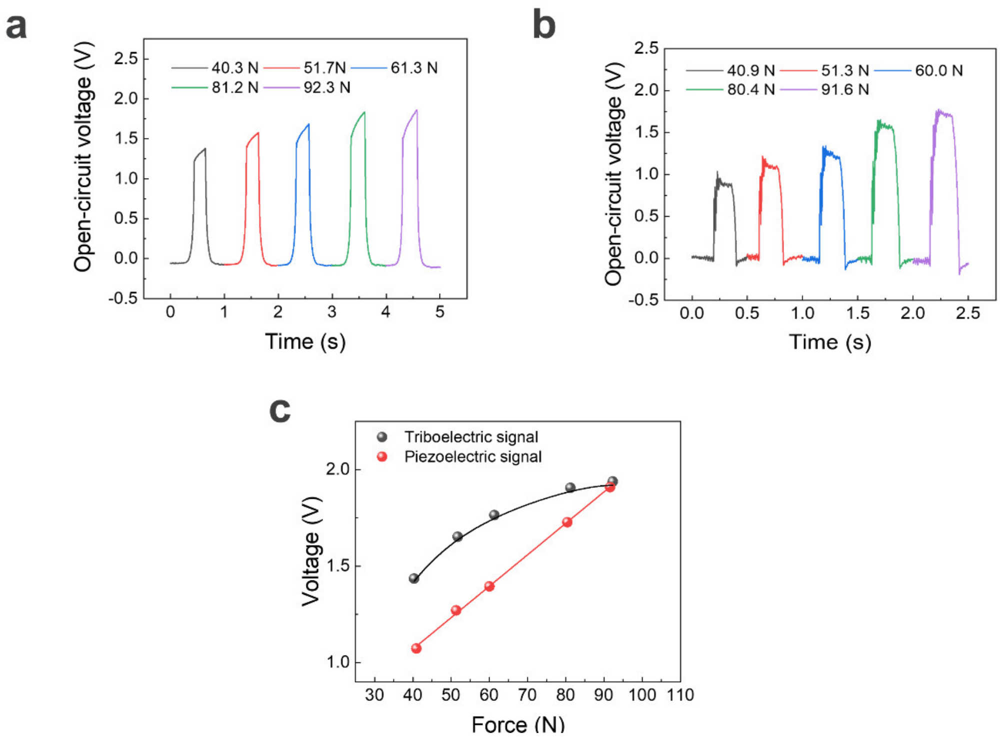

Supplementary Fig. 7 Comparing of the trends of triboelectric signals and piezoelectric signals from 40 N to 90 N (a) Triboelectric signals. (b) Piezoelectric signals. (c) Voltage-force relationships of triboelectric signals and piezoelectric signals.

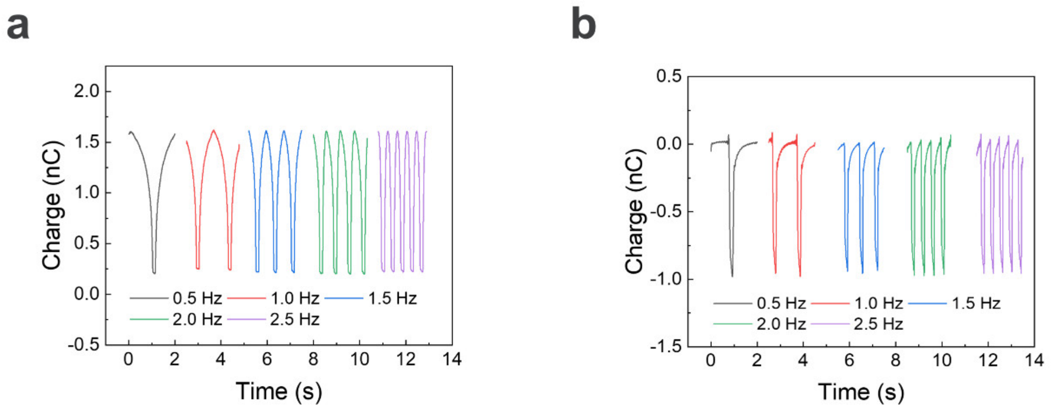

Supplementary Fig. 8 The frequency influence on triboelectric signals and piezoelectric signals. (a) Triboelectric charge transfer and (b) piezoelectric charge transfer are obtained from 0.5 Hz to 2.5 Hz.

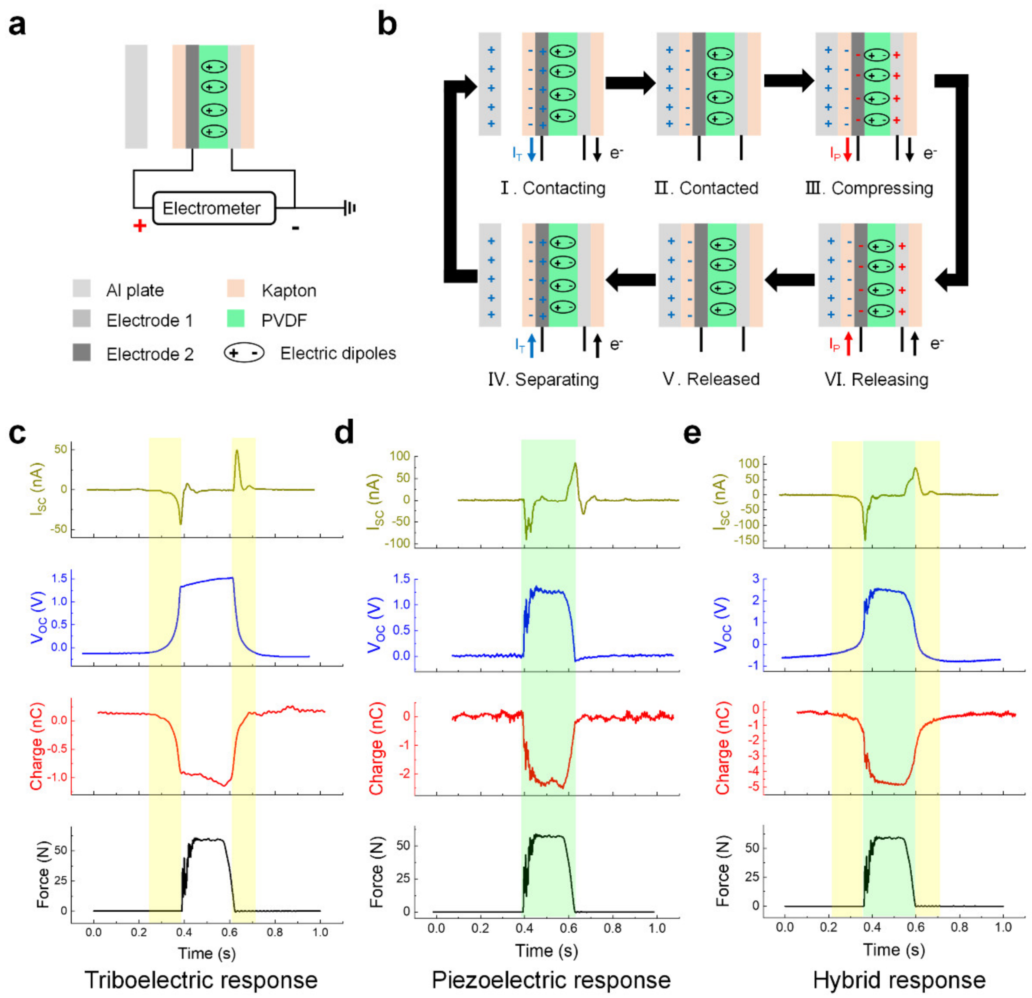

Supplementary Fig. 9 The triboelectric-piezoelectric hybrid output generated by the PVDF-based device with the positive polarization direction. (a) Schematic of measuring the triboelectric-piezoelectric hybrid output in the positive polarization direction of the PVDF film. (b) The signal generation process of hybrid output contains six stages including contacting, contacted, compressing, releasing, released, separating. (c) The sole triboelectric signals (I_{SC}, V_{OC}, and charge) are generated by the PI-based device at 60 N. Sole triboelectric signals are generated in the stages of contacting and separating (light yellow area). (d) The sole piezoelectric signals (I_{SC}, V_{OC}, and charge) are generated by the PVDF-based device with a shielding layer at 60 N. Sole piezoelectric signals are generated in the stages of compressing and releasing (light green area). (d) The triboelectric-piezoelectric hybrid signals (I_{SC}, V_{OC}, and charge) are generated by the PVDF-based device at 60 N. The hybrid signals exist in the stages of contacting, compressing, releasing, and separating.

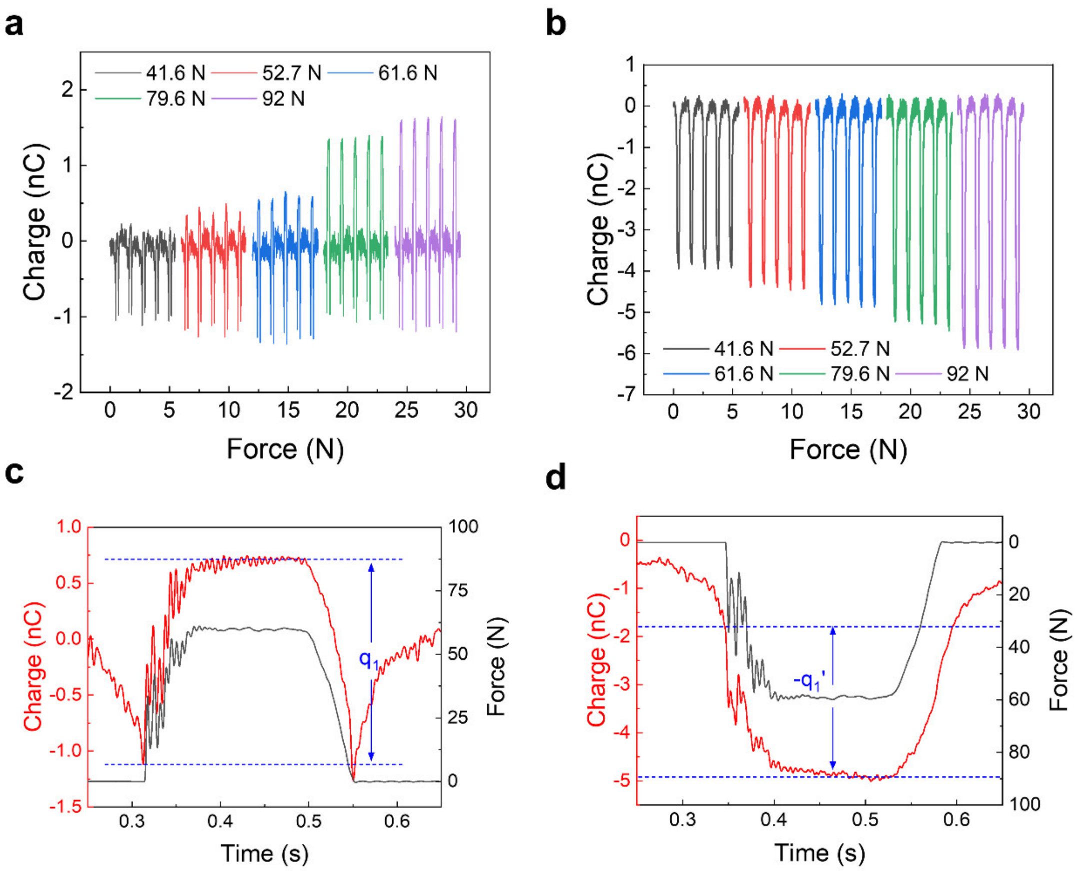

Supplementary Fig. 10 The triboelectric-piezoelectric charge signals are measured in the (a) negative polarization direction and (b) positive polarization direction of the PVDF film under different forces. The piezoelectric charge transfer (c) q_1 and (d) $-q_1'$ are extracted from the hybrid signals (a) and (b), respectively.

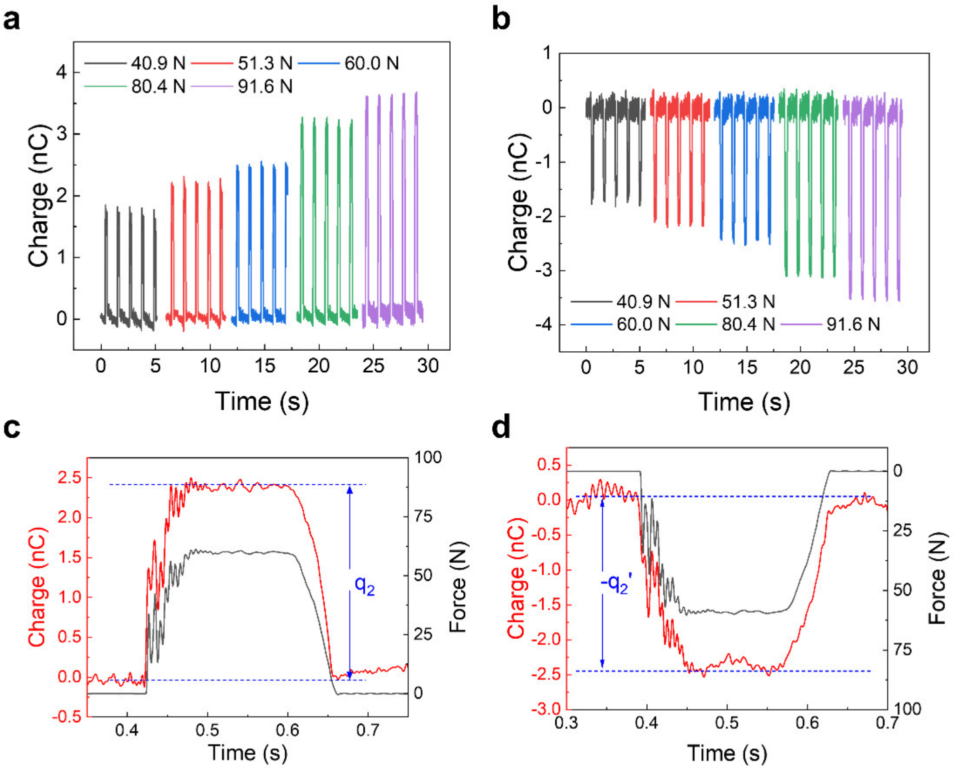

Supplementary Fig. 11 The transferred piezoelectric charges generated by the PVDF-based device with a shielding layer are measured in the (a) forward connection and (b) reverse connection under different forces. The piezoelectric charge transfer (c) q_2 and (d) $-q_2'$ are the peak value in (a) and the minimal value in (b), respectively.

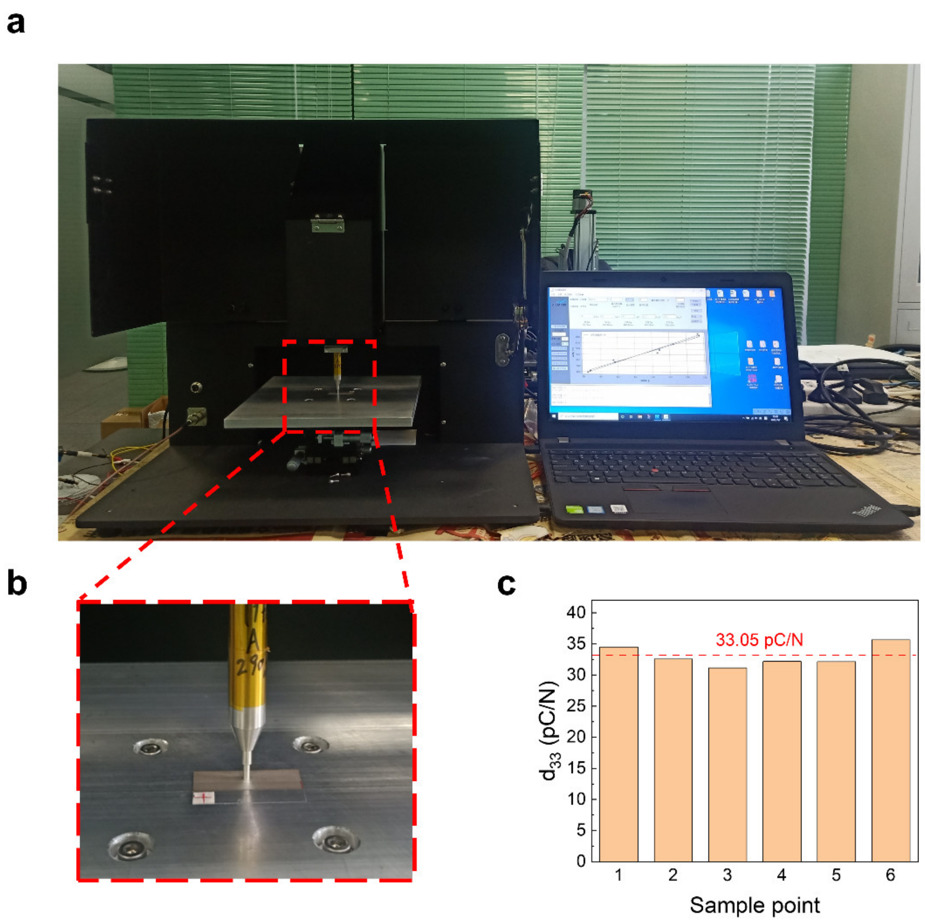

Supplementary Fig. 12 Using a commercial piezoelectric meter to measure the piezoelectric coefficient d_{33} of the PVDF film. (a) Photography of the measurement platform. (b) The force loading process of the PVDF film. (d) The measured d_{33} values from 6 sample points and the average d_{33} of the PVDF film is 33.05 pC/N.

Supplementary Note 1: Calculating the piezoelectric charge transfer using directly extracting (DE) method

The positive piezoelectric charge transfer is q_1 (Supplementary Fig. 10c) and the negative piezoelectric transfer is $-q_1'$ (Supplementary Fig. 10d) in the hybrid output. They are determined by the force curve where the force increases from 0 to the peak, and the relative value between two blue lines is the transferred piezoelectric charge q_1 ($-q_1'$). The average piezoelectric transfer Q_{DE} is:

$$Q_{DE} = \frac{q_1 + q_1'}{2}$$

Supplementary Note 2: Calculating the piezoelectric charge transfer using outside shielding (OS) method

The transferred pure piezoelectric charges obtained from the PVDF-based device with a shielding layer under different forces are shown in Supplementary Fig. 11a, b. The positive piezoelectric charge transfer is q_2 and the negative piezoelectric transfer is $-q_2'$, which are shown in Supplementary Figs. 11c, d, respectively. The average piezoelectric transfer Q_{OS} is:

$$Q_{OS} = \frac{q_2 + q_2'}{2}$$
