# Supplementary material for: A method for quantitatively separating the piezoelectric component from the as-received “Piezoelectric” signal
Source: Nat Commun. 2022 Mar 16;13:1391. doi: 10.1038/s41467-022-29087-w (PMC8927587; doi:10.1038/s41467-022-29087-w)
Supplement: Supplementary file 3 — Description of Additional Supplementary Files [file 41467_2022_29087_MOESM3_ESM.pdf]

Description of Additional Supplementary Files

File Name: Supplementary Movie 1
Description: Video captured the triboelectric charge signal generated by the PI-based device.

File Name: Supplementary Movie 2
Description: Video captured the piezoelectric charge signal obtained from the negative polarization direction of PVDF film, showing a positive direction. The PVDF-based device is covered by a shielding layer.

File Name: Supplementary Movie 3
Description: Video captured the triboelectric-piezoelectric hybrid charge signals, in which these two signals are in the opposite direction.

File Name: Supplementary Movie 4
Description: Video captured the triboelectric-piezoelectric hybrid charge signals, in which these two signals are in the same direction.

File Name: Supplementary Movie 5
Description: Video captured the piezoelectric charge signal obtained from the positive polarization direction of PVDF film, showing a negative direction. The PVDF-based device is covered by a shielding layer.
